# Differential Bacterial Predation by Free-Living Amoebae May Result in Blooms of *Legionella* in Drinking Water Systems

**DOI:** 10.3390/microorganisms9010174

**Published:** 2021-01-15

**Authors:** Mohamed Shaheen, Nicholas J. Ashbolt

**Affiliations:** 1 School of Public Health, University of Alberta, Edmonton, AB T6G 1C9, Canada; mshaheen@ualberta.ca; 2 School of Environment, Science & Engineering, Southern Cross University, Lismore Campus, PO Box 157, Lismore, NSW 2480, Australia

**Keywords:** free-living amoebae, *Legionella*, engineered water systems, Legionnaires’ disease

## Abstract

Intracellular growth of pathogenic *Legionella* in free-living amoebae (FLA) results in the critical concentrations that are problematic in engineered water systems (EWS). However, being amoeba-resistant bacteria (ARB), how *Legionella* spp. becomes internalized within FLA is still poorly understood. Using fluorescent microscopy, we investigated in real-time the preferential feeding behavior of three water-related FLA species, *Willaertia magna, Acanthamoeba polyphaga*, and *Vermamoeba vermiformis* regarding *Legionella pneumophila* and two *Escherichia coli* strains. Although all the studied FLA species supported intracellular growth of *L. pneumophila*, they avoided this bacterium to a certain degree in the presence of *E. coli* and mostly fed on it when the preferred bacterial food-sources were limited. Moreover, once *L. pneumophila* were intracellular, it inhibited digestion of co-occurring *E. coli* within the same trophozoites. Altogether, based on FLA–bacteria interactions and the shifts in microbial population dynamics, we propose that FLA’s feeding preference leads to an initial growth of FLA and depletion of prey bacteria, thus increases the relative abundance of *Legionella* and creates a “forced-feeding” condition facilitating the internalization of *Legionella* into FLA to initiate the cycles of intracellular multiplication. These findings imply that monitoring of FLA levels in EWS could be useful in predicting possible imminent high occurrence of *Legionella*.

## 1. Introduction

*Legionella pneumophila*, a Gram-negative bacterium, indigenous to natural and engineered water systems (EWS) [1,2], has become the number one cause of drinking water-related disease outbreaks in developed countries [3,4,5]. EWS including building water systems and cooling towers, are often reported as the source of exposure to pathogenic *Legionella* [6,7]. *Legionella* coexists in natural and engineered aquatic environments with other bacteria and microscopic eukaryotes like free-living amoebae (FLA), ciliates, and nematodes [8,9,10,11,12], as bacteria serve as a major source of food for FLA and other microeukaryotes [13,14]. *Acanthamoeba, Vermamoeba (Hartmannella)*, and *Naegleria* are the most commonly reported amoebae isolated from different EWS [15,16]. Growth of pathogenic *Legionella* in EWS is considered to occur predominantly via intracellular growth within the susceptible FLA hosts [17,18,19], to very high concentrations considered necessary for causing infections through aerosol exposures [20].

Over millennia, various bacteria that have developed mechanisms against protozoan predation and digestion [21,22,23,24] and even to replicate within predatory host cells [25,26,27] are referred to as amoeba-resistant bacteria (ARB). The increased environmental “fitness” of *Legionella* has been considered to have resulted from amoeba–bacteria interactions [28,29,30]. About one-third of *L. pneumophila*’s genome encodes effector proteins that are required to prevent digestion and to grow intracellularly in amoebae and, coincidently, in human macrophages [31,32]. *L. pneumophila* effectors proteins are functionally redundant (presumably to deal with a wide range of predatory FLA), as elimination of one or many does not affect its overall pathogenic behavior [33,34]. However, the Type IV secretion system called the Icm/Dot system is essential for *L. pneumophila* pathogenicity, to resist digestion by amoebae and to replicate within various host cells [35,36]. However, the question is how the initial interactions occur between *Legionella* and FLA, and if FLA avoid phagocytosing pathogenic *Legionella* in a multispecies aquatic environment [19], what conditions make the FLA phagocytose *Legionella* to enable them to grow intracellularly.

Protozoa, including FLA appear to have recognition mechanisms to choose particular food. While preferential feeding behavior of some protozoa has been studied [37], there is limited information on these selection processes and feeding preferences of amoeba-resistant bacteria (ARB) in multispecies environments. It is apparent that preferential predation by amoebae would affect the biofilm microbial compositions and play an important role in shaping biofilm bacterial communities [38], but the mechanisms and microbial dynamics are not well understood [10,37,39,40]. Certain bacterial species like *Pseudomonas aeruginosa* has been reported to promote *L. pneumophila* uptake by amoeba hosts [41], in contrast, amoeba-symbionts were presumed to prevent intracellular growth of *L. pneumophila* [23], although the mechanisms is unknown. *P. aeruginosa* and *Klebsiella pneumonia* have also been reported to interfere with the growth and persistence of *L. pneumophila* in biofilms [42,43].

Low-level presence of FLA and *Legionella* spp. are expected in natural and EWS [44,45] but it is unclear who (prey or predator) plays the primary role in internalizing the *Legionella* cells in FLA. In addition, how likely *Legionella* cells are picked-up by the amoeba trophozoites to initiate the intracellular growth is unknown. Given the complex interactions of *L. pneumophila* with FLA within water-biofilms, we used fluorescent microscopy to observe in situ the interactions of three FLA species with *L. pneumophila* in the presence of two *E. coli* K12 strains to explore microbial selection processes through amoeba–bacteria interactions. The two very similar *E. coli* strains also would help to determine the precision in this selection process. We used bacteria that expressed different fluorescent proteins (different colors) to facilitate locating (intracellular/planktonic) the cells in-situ and in real time. Overall, this work would help us to understand how the predatory preference of FLA species may cause problematic concentrations of *L. pneumophila* in EWS.

## 2. Materials and Methods

### 2.1. L. pneumophila Culture

*L. pneumophila* Lp02 (ATCC^®^33152) with a pKB127 plasmid containing green fluorescence protein (GFP) (from Ann Karen Brassinga, University of Manitoba, Canada) [46] was grown on BCYE (Buffered Charcoal Yeast Extract) agar plates without antibiotics at room temperature (RT, 22 ± 1 °C) for 5–7 days (to avoid filamentous growth) [47]. *L. pneumophila* cell suspension was prepared by following the procedure described previously [48]. When appropriate, heat-killed *L. pneumophila* (GFP) cells were also used in co-culture experiments. The *L. pneumophila* (GFP) cell suspension in tap water was heated at 75 °C for 10 min in a heat-block to kill them (confirmed by culturing on BCYE agar plate at 37 °C for 7 days).

### 2.2. E. coli Culture

*E. coli* TOP10 (Invitrogen) cells were transformed with pBad-EBFP2 plasmid (provided by Prof. Robert E. Campbell, University of Alberta) to express a blue fluorescent protein. The other *E. coli* K-12 strain (MG 1655, genotype: F^−^, λ^−^, rph-1) contains the plasmid (pTV-mCherry) expressing a red fluorescent protein (provided by Dr. Tracy Raivio, University of Alberta). These two *E. coli* strains were grown on LB agar plates at 37 °C for 24 h. Cell suspension of each bacterium was prepared in filtered-sterile tap water, and the cell concentration was estimated by checking the optical density at 600 nm and confirmed by culture method.

### 2.3. Amoebae Culture

*W. magna* (ATCC^®^50035) was grown in Serum Casein Glucose Yeast Extract Medium (SCGYEM) at RT for 3 d in 25-cm^2^ cell-culture flasks to obtain trophozoites. *A. polyphaga* (ATCC^®^30461) and *V. vermiformis* (ATCC^®^50237) were grown separately at RT in Peptone Yeast Extract Glucose (PYG) medium for 2 days in 25-cm^2^ cell-culture flasks for trophozoites. The trophozoites of each amoeba species were harvested individually by centrifugation at 400× *g* for 10 min, washed three times with filtered-sterile, dechlorinated tap water, and re-suspended in the same medium to a concentration of approximately 10^5^ trophozoites mL^−1^.

### 2.4. Amoeba–Bacteria Co-Culture

All bacterial strains were mixed together at equal concentrations and added to individual FLA species to make a final ratio of bacteria:trophozoites of 300:1 in filtered-sterile tap water. *L. pneumophila* cells were also mixed separately with different FLA species trophozoites at a ratio of 100:1 in filtered-sterile tap water. Five milliliters of these bacteria-amoeba suspensions were dispensed in 25-cm^2^ cell-culture flasks (about 4.0 × 10^5^ trophozoites per flask). The mixed bacterial suspension was also dispensed in 25-cm^2^ cell-culture flasks and diluted to 5 mL to have a final concentration of 4.0 × 10^7^ cells of individual strains in sterile tap water in each flask (as an amoeba-negative control) to observe whether different bacterial species has any effect on each other. Heat-killed *L. pneumophila* cells were added with viable *E. coli* cells in amoeba co-culture in a similar experimental setup. The experiments were undertaken at RT and in triplicate.

### 2.5. Fluorescent Microscopy and Image Processing

The amoebae–bacteria co-cultures were observed at multiple time points (at 5 and 30 min and 24, 48, 72, and 96 h of incubation) to check the amoebae–bacteria interactions and physical locations (intracellular in amoeba trophozoites and extracellular outside the trophozoites in the medium) of different bacteria using an EVOS Cell Imaging Systems (Thermo Fisher Scientific). When required (for microscopy and before antibiotic treatment), the co-cultures were washed with the filtered-sterile tap water, to reduce the planktonic bacterial cell number by gently changing the water without interrupting the surface-adhered amoeba trophozoites to observe better the intracellular (food vacuoles) location of the bacteria. Random bright field and fluorescent images were taken from each 25-cm^2^ cell-culture flasks at different time points. The images were further processed using ImageJ software (version 1.52e), if required and the number of trophozoites with and without internalized bacteria (different strains) in each field of view was counted.

### 2.6. Determining the Intracellular Bacteria

To enumerate the intracellular bacteria, only two bacterial strains (*L. pneumophila* and *E. coli* TOP10) were mixed together at equal concentrations and added to *W. magna* (ATCC^®^30035) trophozoites to make a final ratio of bacteria:trophozoites of 200:1 in filtered-sterile tap water in 25-cm^2^ cell-culture flasks and incubated at RT. At different time points (0.5, 24, 48, and 96 h) of the co-culture, the medium was aspirated gently from the flasks (in duplicate) and replaced once with 3 mL of sterile water to reduce the planktonic bacterial cell number (as much as possible without disturbing the adhered trophozoites to the flask bottom). Three milliliters of filtered-sterile tap water containing gentamicin (200 µg/mL) was added to the cell-culture flask containing amoeba trophozoites with mostly internalized bacteria and incubated at RT for 1 h to kill the remaining planktonic bacterial cells. The trophozites were harvested from the flasks after 1 h and re-suspended in 1.5 mL water in 2-mL tubes. The trophozoites were washed three times by centrifuging at 2000× *g* and resuspending in water. Finally, the trophozoites were lysed by passing through (back and forth) a 23-gauge needle five times to release the internalized bacteria. Appropriate dilutions of these cell suspensions were plated (spread plate technique) on BCYE plate with antibiotics (Polymyxin, Cycloheximide and Vancomycin) and LB agar plate to determine the number of *L. pneumophila* and *E. coli* TOP10 cells, respectively. The plates were incubated at 37 °C overnight for *E. coli* TOP10 and 5 days for *L. pneumophila.*

### 2.7. Statistical Analysis

The number of trophozoites were counted from 12 random fields of view photo taken under different fluorescent and bright field channels for visualizing internalized bacteria (studied bacteria produce Green, Red, and Blue fluorescent proteins). Student t-test was carried out to compare the feeding preference of the amoeba trophozoites for different bacterial strains.

## 3. Results

### 3.1. Differential Feeding Preference of W. magna

**Figure 1:**
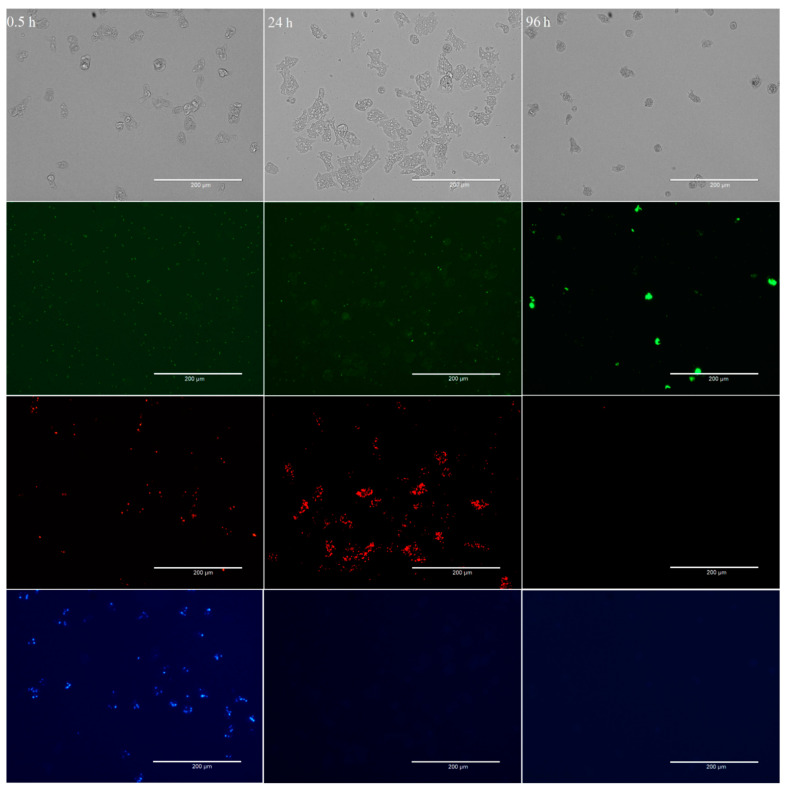
Preferential feeding on bacteria by *W. magna* at RT at different time points (0.5, 24, and 96 h) of co-culture. The four images (from top to bottom) represent the same field of view under different fluorescent light channels, Monocolor transmission light channel, Green fluorescent channel to observe GFP-*L. pneumophila*, Texas-Red channel for mCherry-*E. coli* MG1655, and DAPI channel for BFP-*E. coli* TOP10. The clusters of color dots in images indicate the presence of different intracellular bacteria in the food vacuoles of *W. magna* trophozoites. The scattered color dots (smaller in size) indicate planktonic bacterial cells in the medium.

Large number of *E. coli* cells were found accumulated in the food vacuoles of *W. magna* trophozoites as early as 5 min of co-culturing them in sterile tap water at RT. Even though *W. magna* fed on both the *E. coli* strains, there was a clear preference (visual observation of microscopic images) for *E. coli* TOP10 cells over *E. coli* MG1655 (as determined by the apparent number of food vacuoles containing bacteria and the intensity of fluorescence) (Figure 1).

**Figure 2:**
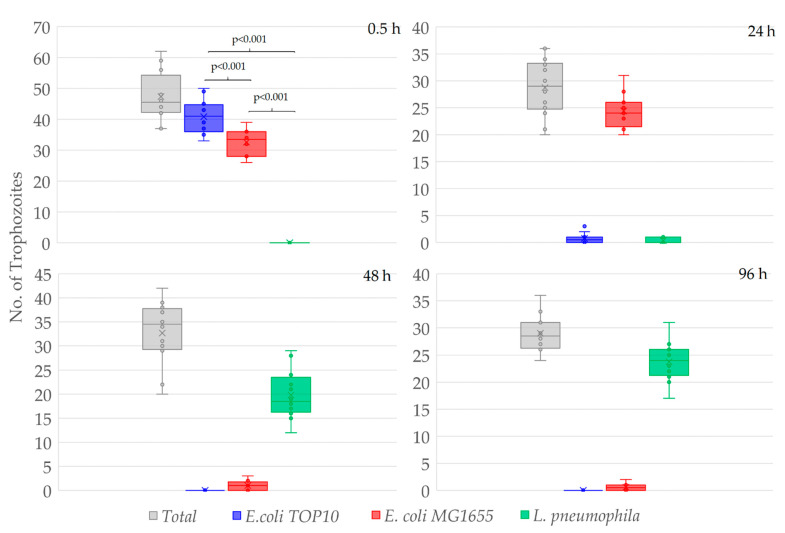
The number of *W. magna* trophozoites containing *E. coli* TOP10, *E. coli* MG1655, and *L. pneumophila* cells at different time points of co-culture. The number of trophozoites were counted from four fields of view of three replicates (total 48 images/ time point), the bars represent standard deviations of the mean and the box represent 25–75 percentiles. The *t*-test indicates significant differences in prey preference (*p* < 0.001).

After 0.5 h of co-incubation, 87.1 ± 9.0% of the total *W. magna* trophozoites contained *E. coli* TOP10 cells, 69.7 ± 9.0% *E. coli* MG1655 and none contained *L. pneumophila*, despite being present in equally high numbers in close proximity to the amoeba trophozoites (Figure 2). Although both the *E. coli* strains were present in the same trophozoites, the number of food vacuoles containing *E. coli* TOP10 cells was much higher than that contained the *E. coli* MG1655 cells.

After 24 h of co-incubation, no *E. coli* TOP10 cells were observed in the medium and hardly any in the food vacuoles of the trophozoites (*E. coli* TOP10 cells were digested), however, *E. coli* MG1655 cells were numerous within food vacuoles. After 24 h, 2.8 ± 2.8% of the total amoeba trophozoites contained *E. coli* TOP10 cells, 87.0 ± 10.1% contained *E. coli* MG1655, and 1.5 ± 1.9% contained *L. pneumophila*. Hence, *E. coli* MG1655 appeared to be the second preferred food by *W. magna* under the study conditions and exhibited some resistance to amoeboid digestion as compared to *E. coli* TOP10, but both were eventually digested by the amoeba trophozoites within 48–72 h of co-culture. After 48 and 96 h of co-culture no *E. coli*, TOP10 cells were observed within trophozoites, only a few (3.5 ± 3.1% and 2.1 ± 2.1%, respectively) contained *E. coli* MG1655 and most (61.0 ± 8.6% and 81.8 ± 6.5%, respectively) contained *L. pneumophila*. The intracellular concentrations of different bacteria at different time points confirmed the preferential feeding of bacteria by *W. magna* (Figure S1). *W. magna* also showed similar preferential feeding behavior with heat-killed *L. pneumophila* when provided in presence of the *E. coli* strains (Figure S2).

**Figure 3:**
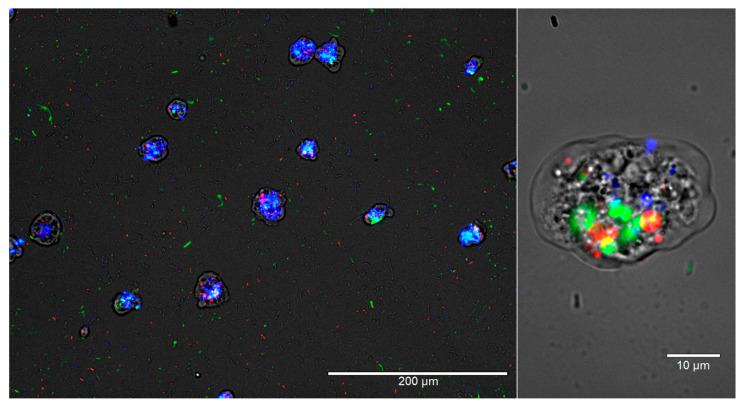
Intracellular *L. pneumophila* interferes with *W. magna*’s digestion process. *W. magna* and *L. pneumophila* were co-cultured at RT, and after 24 h, *E. coli* TOP10 and *E. coli* MG1655 were added to the culture. The images represent overlaid images of the same field of view under four light channels, Monocolor transmission light channel, Green fluorescent channel, Texas-Red channel, and DAPI channel at 200× and 1000× magnification after 26 h of co-culture. *L. pneumophila* cells are green, *E. coli* MG1655 cells are red, and *E. coli* TOP10 cells are blue in color.

Thus, *W. magna* only appeared to phagocytose *L. pneumophila* when other bacterial strains (*E. coli*) were unavailable in the co-culture due to prior predation. After 72 h, intracellular growth of *L. pneumophila* was observed in many trophozoites but not in all that contain *L. pneumophila* in the food vacuoles. None of the *E. coli* strains produced any apparent adverse effect on *W. magna* growth and activity (i.e., all demonstrated regular gliding movement (Supplementary Information Video S3 and Video S4). The gliding movement of the trophozoites with fluorescent food vacuoles also confirmed the intracellular status of the targeted cells. *W. magna* even avoided heat-killed *L. pneumophila* when present with the two *E. coli* strains in the same culture. However, *W. magna* phagocytosed *L. pneumophila* within 24 h of co-culture in sterile tap water, when provided as a single species bacterial prey. Interestingly, trophozoites that had recently acquired *L. pneumophila* also subsequently phagocytosed *E. coli* strains upon adding them to the culture (Figure 3 and Supplementary Information Video S5), but could not digest them as quickly as they could without having the intracellular *L. pneumophila*.

Hence, internalized *L. pneumophila* seemed to interfere with the overall digestion process of trophozoites and may lead to long-term intracellular persistence of the bacteria without initiating active intracellular growth at RT. All the bacterial species remained fluorescent without losing their number in amoeba-negative culture up to 4 days in sterile tap water at RT. No adverse effect was observed on each other by the studied bacterial species (Figure S6).

### 3.2. Interactions of A. polyphaga and V. vermiformis with Bacteria

**Figure 4:**
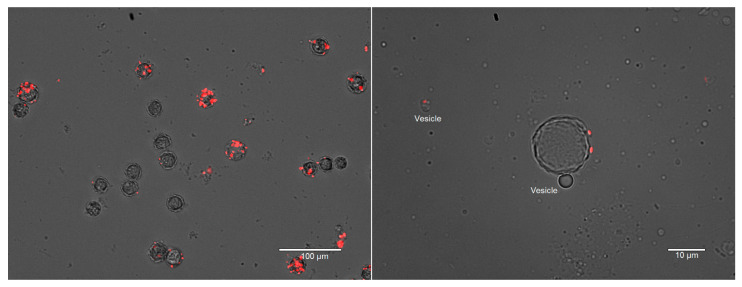
Intracellular locations of *E. coli* MG1655 (red) in *A. polyphaga* cysts at 96 h of co-culture. Many of the cysts did not contain any *E. coli* MG1655 cells, which indicate that the digestion may depend on the intracellular load of the bacterial cells and encystation time. Releasing of vesicle during encystation and vesicle-containing *E. coli* MG1655 after 48 h of co-culture (Right image).

*A. polyphaga* and *V. vermiformis* exhibited a higher preference for both *E. coli* strains when all three bacterial species were present in co-culture in sterile tap water or PAGE’s saline at RT (Figures S7 and S8). However, *A. polyphaga* did not avoid *L. pneumophila* as strongly as *W. magna* and *V. vermiformis* did. In tap water *A. polyphaga* tend to form cyst within 48 h and therefore, PAGE’s saline was used. *E. coli* TOP10 was found to be phagocytosed and mostly digested within 24 h by *V. vermiformis*, therefore hardly any *E. coli* TOP10 cells were found in the medium as well as in the food vacuoles of the trophozoites after 24 h of co-culture. However, due to presence of internalized *L. pneumophila* in *A. polyphaga* trophozoites, *E. coli* cells were still observed in the food vacuoles after 24 h. Due to the rapid encystation of *A. polyphaga* and *E. coli* MG1655′s moderate resistance to the amoeboid digestion, it stayed in the cysts, most likely in between the two outer layers of the cysts (Figure 4). Releasing of vesicles during encystation was also observed in *A. polyphaga*, as reported previously for the protozoa *Giardia* [49,50]. Both the amoebae phagocytosed *L. pneumophila* when provided as a single culture and supported intracellular growth.

### 3.3. Amoebae–Bacteria Interactions

When all three species of the amoebae (trophozoites) were co-cultured with the studied bacterial species in sterile tap water at RT, the amoebae phagocytosed and digested the *E. coli* strains first before effectively engulfing the *L. pneumophila*. Of particular note, co-presence of the amoebae species did not appear to affect their feeding preferences. However, in water *A. polyphaga* and *V. vermiformis* underwent encystation earlier than *W. magna*, resulting in *E. coli* MG1655 cells being entrapped in cysts (Supplementary Information Video S9 and Video S10), as previously reported for *L. pneumophila* [51]. *W. magna* was also observed to phagocytose cysts of the other amoeba species present under the studied conditions (Figure S11).

## 4. Discussion

It is well known that FLA support the intracellular growth of pathogenic *Legionella* and other similar opportunistic water-based human pathogens. In fact, the ability to grow within amoebae has led to the evolution of *L. pneumophila* as a human pathogen [39]. However, we have very limited information on how pathogenic *Legionella* interacts with FLA in its natural water-biofilm environment. Our previous study suggested that *Legionella* might not be the preferred prey for *W. magna* within drinking water-biofilms [19]. This study provided visual evidence to support earlier observation by showing the interactions of different FLA species and *L. pneumophila* in the presence of other bacteria in real-time in situ. Accumulation of large numbers of *E. coli* cells and no *Legionella* in food vacuoles of *W. magna* trophozoite within 5 min of co-incubation indicated a very fast, effective, and precise recognition mechanisms since the bacteria were present in a homogenous equally high concentration suspension. The sequential feeding order of the two *E. coli* strains and *Legionella* by *W. magna* also indicated that amoeba played the active role in recognizing food through a highly selective manner. Although the two *E. coli* strains (TOP10 and MG1655) are very similar and originated from a common ancestor (*E. coli* K12), it was surprising that *W. magna* distinguished even between these two strains. The higher tolerance of *E. coli* MG1655 to amoeboid digestion implied that the resistance to digestion was from the bacterial side. *W. magna* was reluctant to feed on *L. pneumophila* until other options were limited, therefore, amoebae–bacteria interactions increased the relative abundance of *L. pneumophila* and created a “forced-feeding” situation to insist the amoebae to phagocytose *Legionella* [19]. Phagocytosing of *L. pneumophila* by *A. polyphaga* and *V. vermiformis* but not by *W. magna* in the presence of other bacteria indicated that the recognition systems for food were different among the amoeba species and might not be very stringent in all amoebae, as no apparent effect of non-*Legionella* bacteria on the uptake of *L. pneumophila* by *A. castellanii* and *N. lovaniensis* was reported earlier [41].

Further research is required to characterize FLA-*L. pneumophila* interactions in complex natural environments where other organisms and biofilms are present. Nonetheless, it is apparent from the current study that FLA may recognize extracellular chemicals and/or virulence-associated surface markers of *L. pneumophila* since *W. magna* was even ‘unwilling’ to phagocytose heat-killed *Legionella* in the presence of *E. coli*. Chemotaxis movement of amoeba toward certain bacterial cell lysate supports this observation [37]. Amoebae phagocytosed *L. pneumophila* when provided as the only option—suggested a “forced-feeding” condition when FLA had no choice but to feed on *L. pneumophila*, despite being detrimental to them. Larger amoeba trophozoites also phagocytosed other amoeba cysts, which could be another example of “forced-feeding.” Thus, this study strengthens our hypothesis that selection of *L. pneumophila* through preferential feeding of FLA creates conditions when *L. pneumophila* becomes the main available food and “forced-feeding” by FLA leads to *L. pneumophila*’s ultimate rapid growth in water [19].

The prolong presence of undigested *E. coli* in trophozoites containing *L. pneumophila* indicates that *L. pneumophila* actively interferes with the amoeboid digestion process. Hence, low intracellular concentrations of *L. pneumophila* may render the amoeba cell a reservoir of the bacteria by potentially “intoxicating” the amoeba trophozoites and impairing their digestion process. Similarly, the trapped bacteria within the amoeba cysts also could serve as a source of contamination when the cysts germinate. Moreover, the cysts protect the internal bacteria from the harsh environment and chemical disinfectants [52]. These cysts with *L. pneumophila* explain the recurrent LD outbreaks within hospital plumbing systems with clonal strains over decades [53,54,55,56]. Vigorous treatment of water systems in case of LD outbreak may remove the planktonic or biofilm-associated cells to some extent but leave behind the cysts with bacteria, which may act as a source for subsequent LD outbreaks [57].

Although the Icm/Dot, the type IV secretion system of *L. pneumophila*, is essential for replication within the amoebae and kill them [35,36], no adverse effects on *E. coli* suggested that the Icm/Dot system has no antibacterial activity as reported for type VI secretion system of *V. cholerae* [58]. The type VI secretion system of *V. cholera*e is also required to kill the protozoa, *Dictyostelium discoideum* [59].

Overall, this study supports our previous hypothesis that preferential feeding of FLA might be the driving force for rapid growth of pathogenic *Legionella* and other ARB in EWS. Although it is well known that the pathogenic *L. pneumophila* can grow intracellularly and disperse in water as free or vesicle-bound cluster of cells [48,60,61], the current work has described a possible mechanism of attaining critical concentrations of *L. pneumophila*. Ultimately, this work helped to understand the ecological perspective of *L. pneumophila*’s growth in EWS where it is usually present at very low concentrations. FLA’s reluctance to graze on *L. pneumophila* in the presence of non-ARB also suggests that a probiotic approach could work to control pathogenic *Legionella*’s growth in water systems. Ecological interactions such as competition, antagonism, and obligate parasite–host relationships have been described for potential targets for probiotic control of opportunistic pathogens in EWS [62]. In fact, upstream microbiota has been described to have a profound effect on the downstream biofilm bacterial compositions in water pipes [63,64].

## 5. Conclusions

Since intracellular multiplication in FLA is the major means for *L. pneumophila*’s growth, understanding the ecology of *L. pneumophila*, especially its interactions with FLA are fundamental to better management of water in EWS and to ensure public health safety. This study provided visual evidence of how FLA–bacteria interactions could lead to the problematic growth of pathogenic *Legionella* in EWS. Hence, the current monitoring of *L. pneumophila* without any consideration on FLA appears to be a weakness in water quality monitoring for EWS. Since FLA appears to be a major driving force for bacterial community shifts towards selection of opportunistic water-based pathogens, more research on other FLA and opportunistic pathogens like nontuberculous mycobacteria and *Pseudomonas* spp. is required to develop generalized approaches for monitoring and control of these environmental pathogens.

## Data Availability

The data presented in this study are available on request from the corresponding author. All the image data have not been stored in publicly available domain but additional images and videos are provided in the supplementary information.

## Supplementary Materials

The following are available online at https://www.mdpi.com/2076-2607/9/1/174/s1.
