# Supplementary material for: Differential Bacterial Predation by Free-Living Amoebae May Result in Blooms of Legionella in Drinking Water Systems
Source: Microorganisms. 2021 Jan 15;9(1):174. doi: 10.3390/microorganisms9010174 (PMC7829821; doi:10.3390/microorganisms9010174)
Supplement: Supplementary file 1 [file microorganisms-09-00174-s001.zip › Supplementary information.docx]

Differential bacterial predation by free-living amoebae may result in blooms of *Legionella* in drinking water systems

## Mohamed Shaheen ^1^ and Nicholas J. Ashbolt ^2,^*

## Supplementary Information


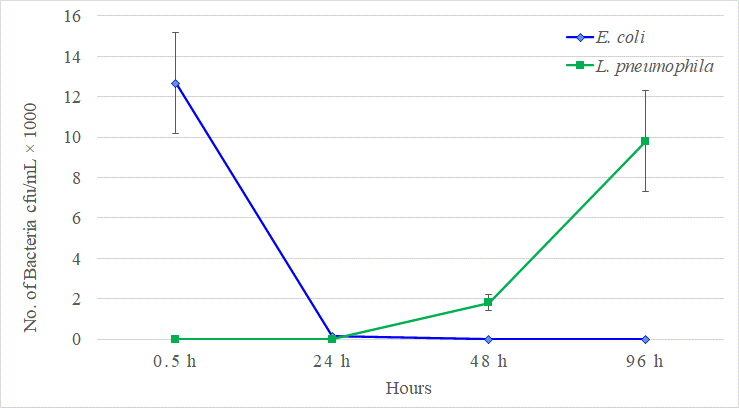


Figure S1. Intracellular concentrations of *L. pneumophila* and *E. coli* TOP10 cells within *W. magna* trophozoites at different time points. At 0.5 h the intracellular bacteria are mostly *E. coli* but after 24 h it is almost absent on the other hand at 0.5 h there is hardly any intracellular *L. pneumophila* but after 48 h it become the main intracellular bacteria in *W. magna* trophozoites.


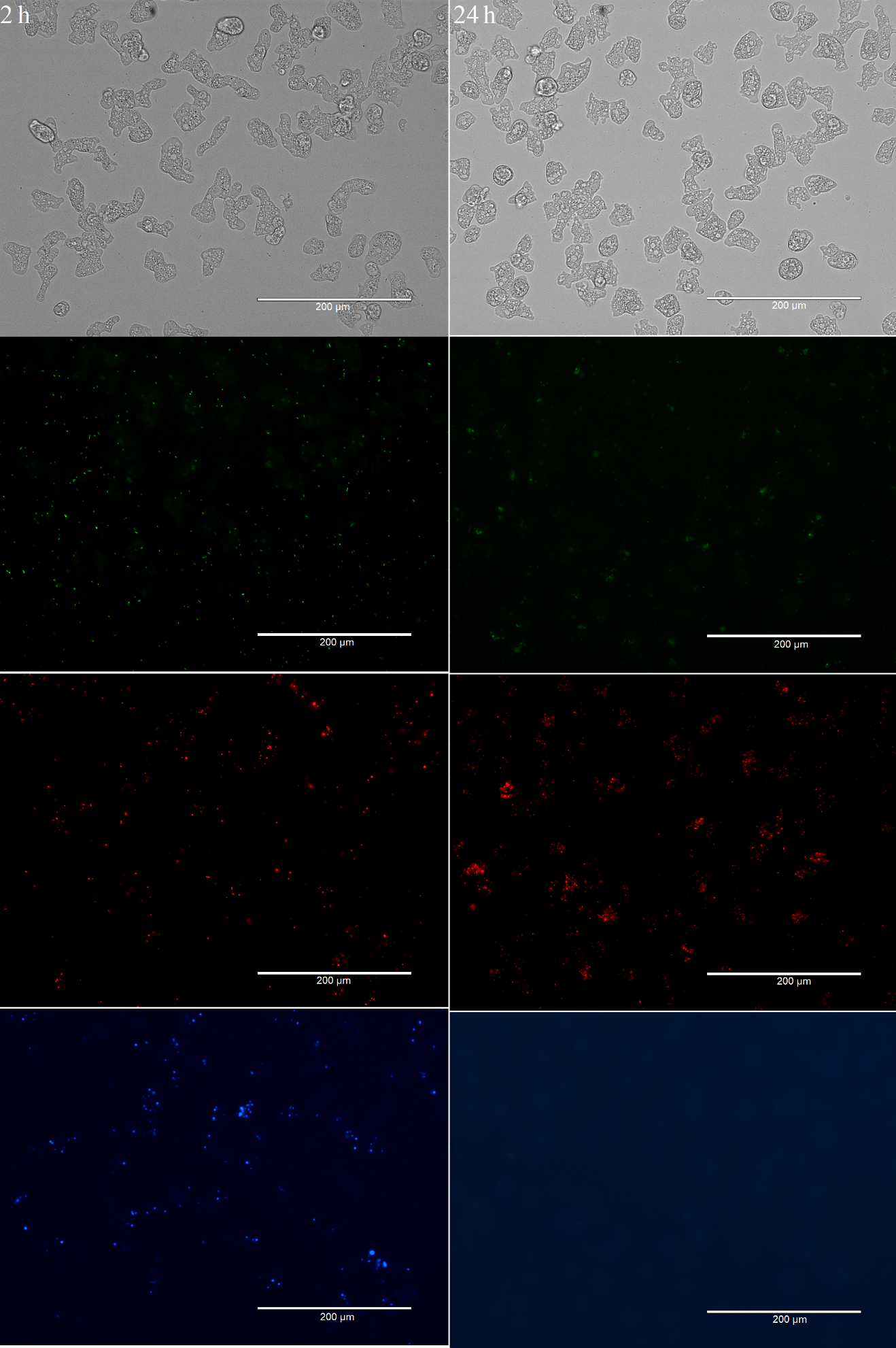


Figure S2. Co-culture of *W. magna* at RT with heat killed *L. pneumophila* in presence of the *E. coli* strains at different time points (2 and 24 h). The four images (from top to bottom) represent the same field of view under different fluorescent light channels, Mono-color transmission light channel, Green fluorescent channel to observe heat killed GFP-*L. pneumophila*, Texas-Red channel for mCherry-*E. coli* MG1655, and DAPI channel for BFP-*E. coli* TOP10 respectively.


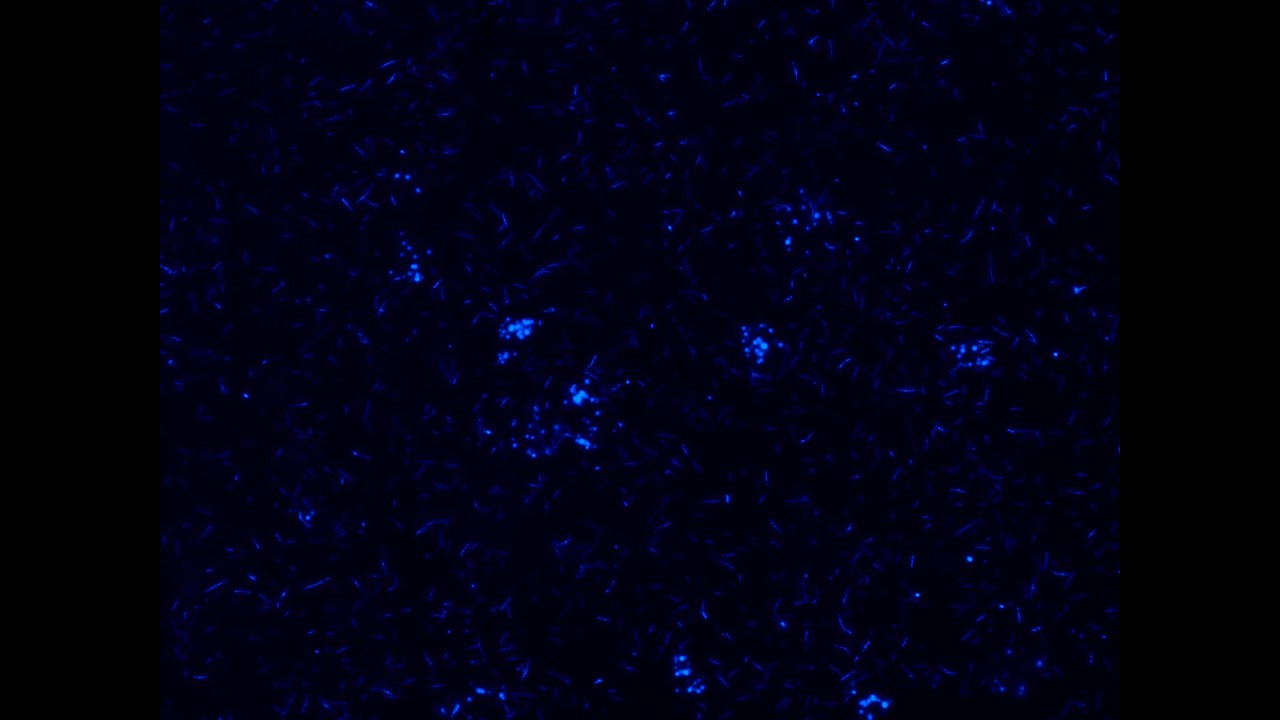


Video S3. Planktonic and intracellular locations (in food vacuoles of *W. magna* trophozoites) of BFP producing *E. coli* TOP10 under DAPI florescent channel*.*


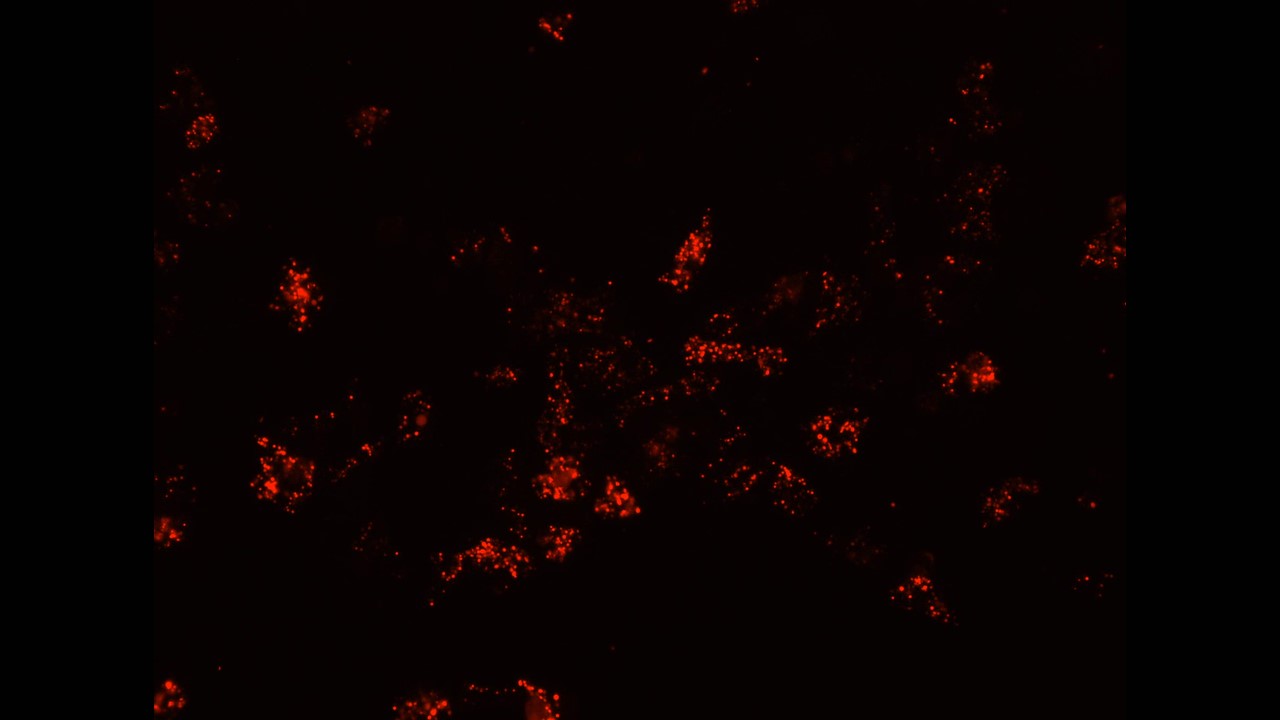


Video S4. Planktonic and intracellular locations (in food vacuoles of *W. magna* trophozoites) of mCherry-*E. coli* MG1655 under Texas-Red florescent channel.


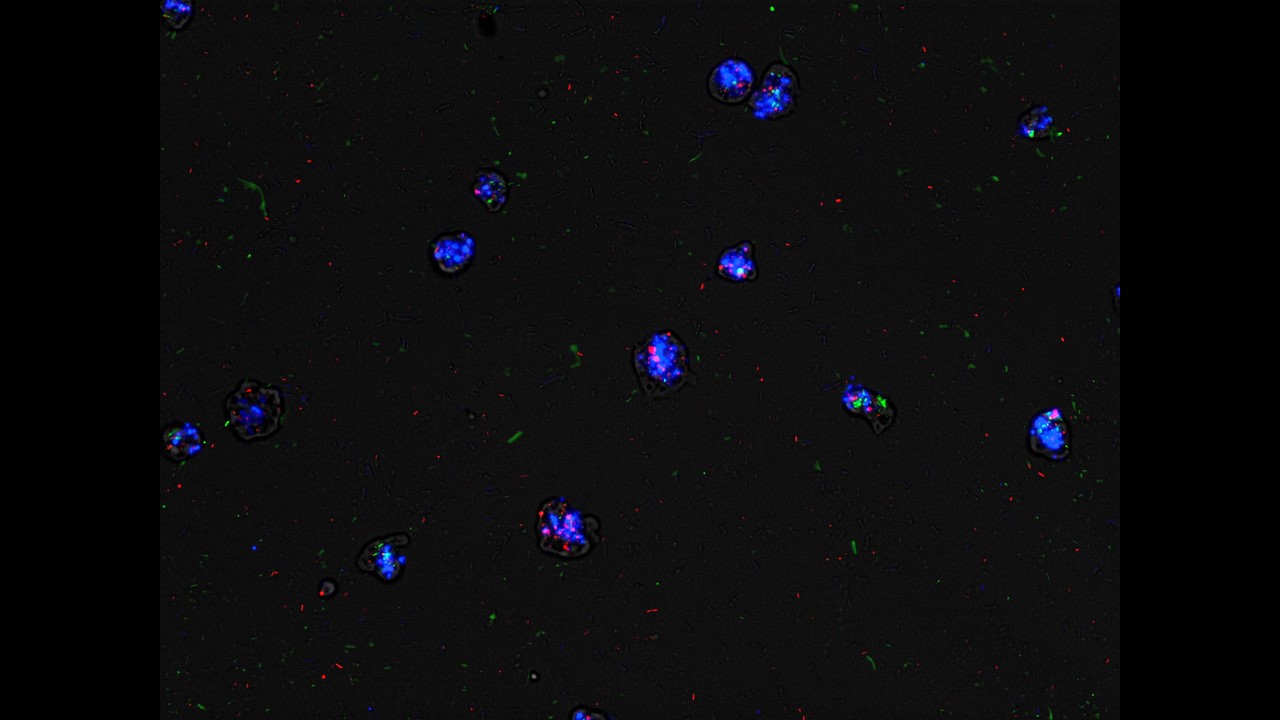


Video S5. Planktonic and intracellular locations (in food vacuoles of *W. magna* trophozoites) of all three bacteria (*L. pneumophila* in green, *E. coli* MG1655 in red and *E. coli* TOP10 in blue). The trophozoites were co-culture with *L. pneumophila* only and after 24 h *E. coli* MG1655 and *E. coli* TOP10 were added to the same co-culture. The video represents a series of overlaid images taken simultaneously under four channels (Mono-color transmission light channel to observe the amoeba trophozoites, Green fluorescent channel to observe GFP-*L. pneumophila*, Texas-Red channel mCherry*-E. coli* MG1655*,* DAPI channel for BFP- *E. coli* TOP10.)


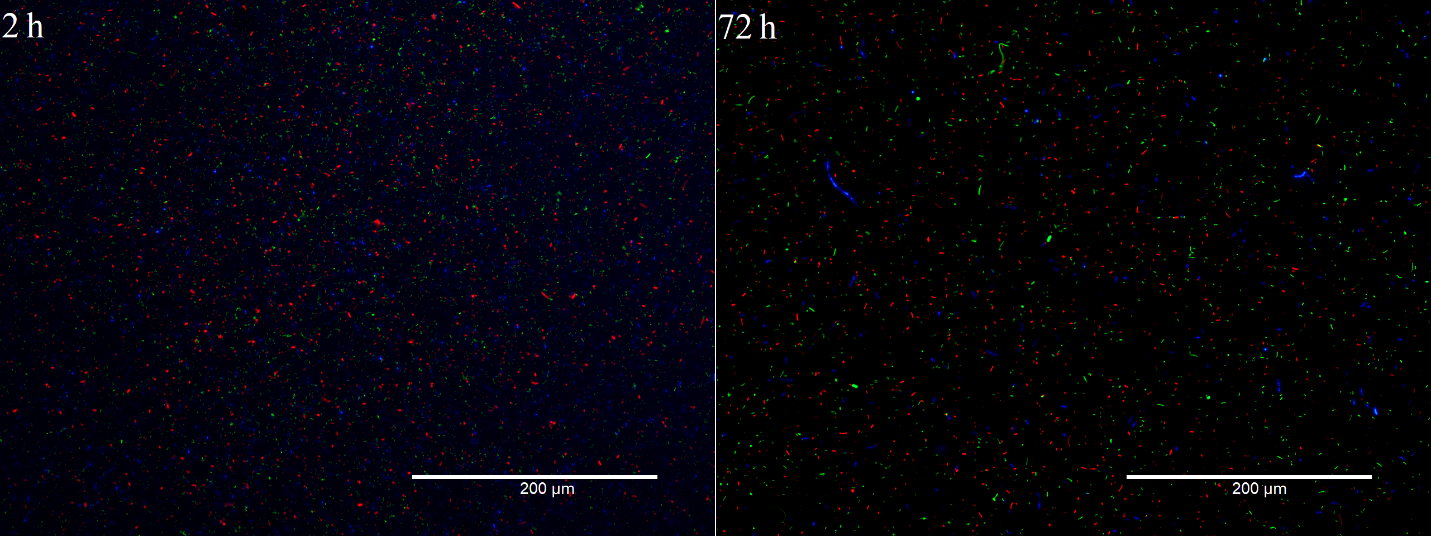


Figure S6. Co-incubation of *L. pneumophila*, *E. coli* MG1655 and *E. coli* TOP10 cells at RT in sterile tap water after 2h (left) and 72 h (right). No adverse effects were observed on any bacterium due their co-presence.


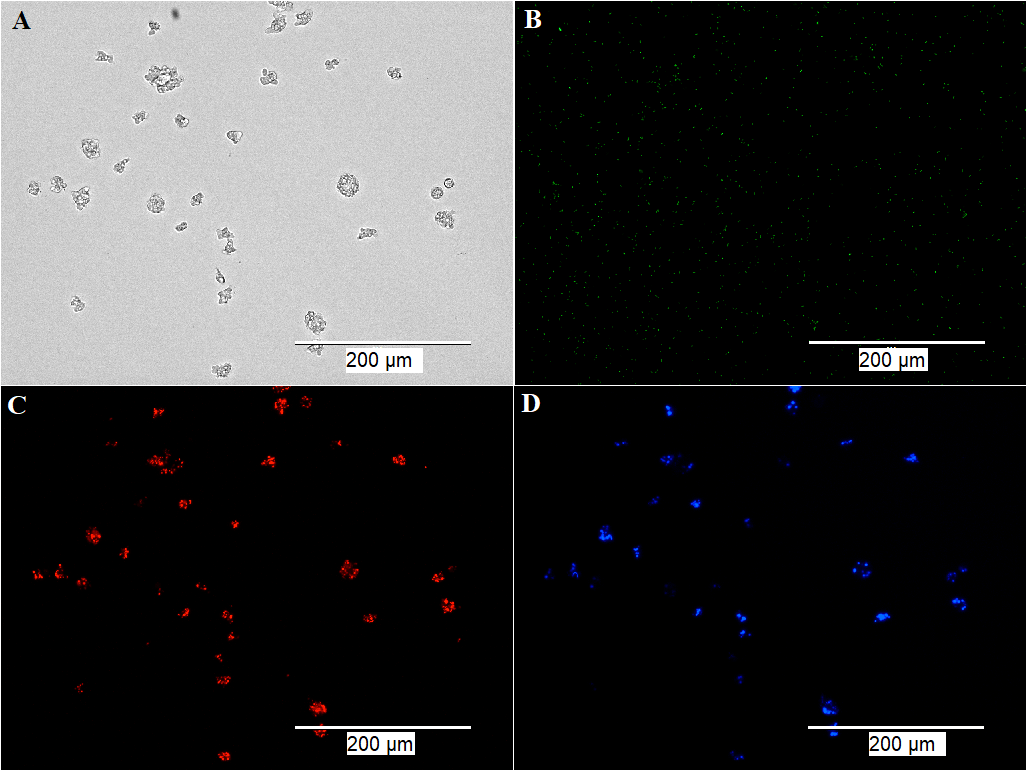


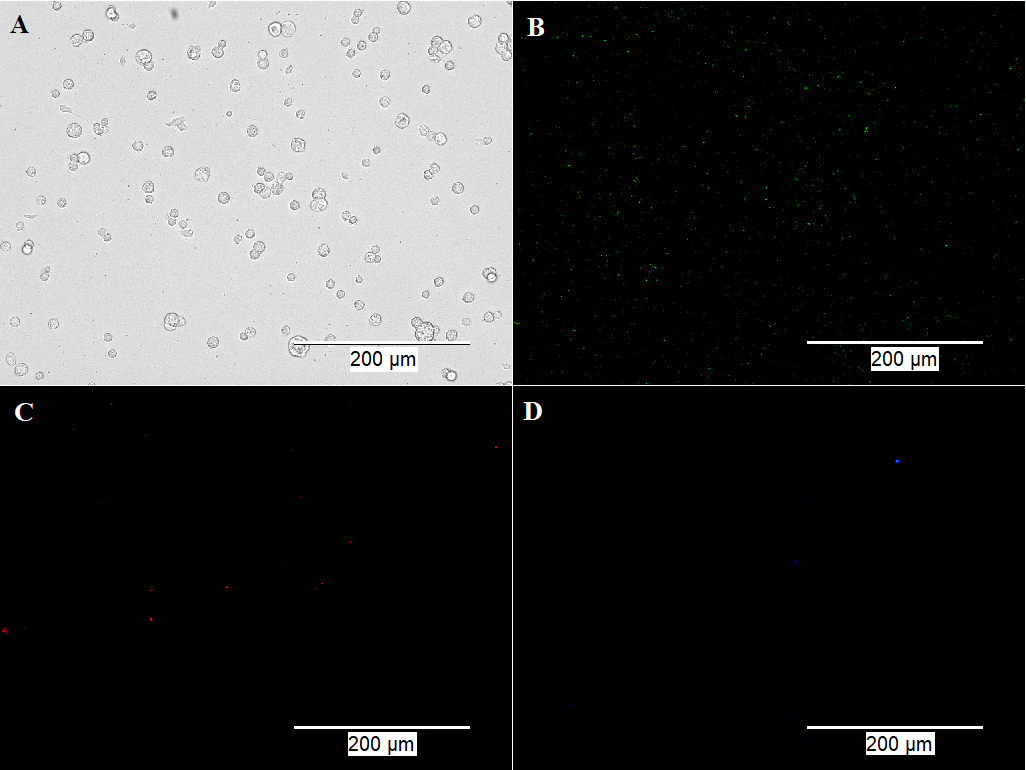


Figure S7. Preferential feeding on bacteria by *V. vermiformis* at RT at 2 h (Top) and 24 h (Bottom) of co-culture. The four images represent the same field of view under different fluorescent light channels. A. Mono-color transmission light channel, B. Green fluorescent channel to observe GFP-*L. pneumophila* C. Texas-Red channel mCherry-*E. coli* MG1655 D. DAPI channel for BFP-*E. coli* TOP10. The cluster of Red cells in image C and Blue cells in image D indicate the presence of *E. coli* MG1655 and *E. coli* TOP10 in the food vacuoles of *V. vermiformis* trophozoites. The scattered green cells represent planktonic *L. pneumophila* cells in the medium.


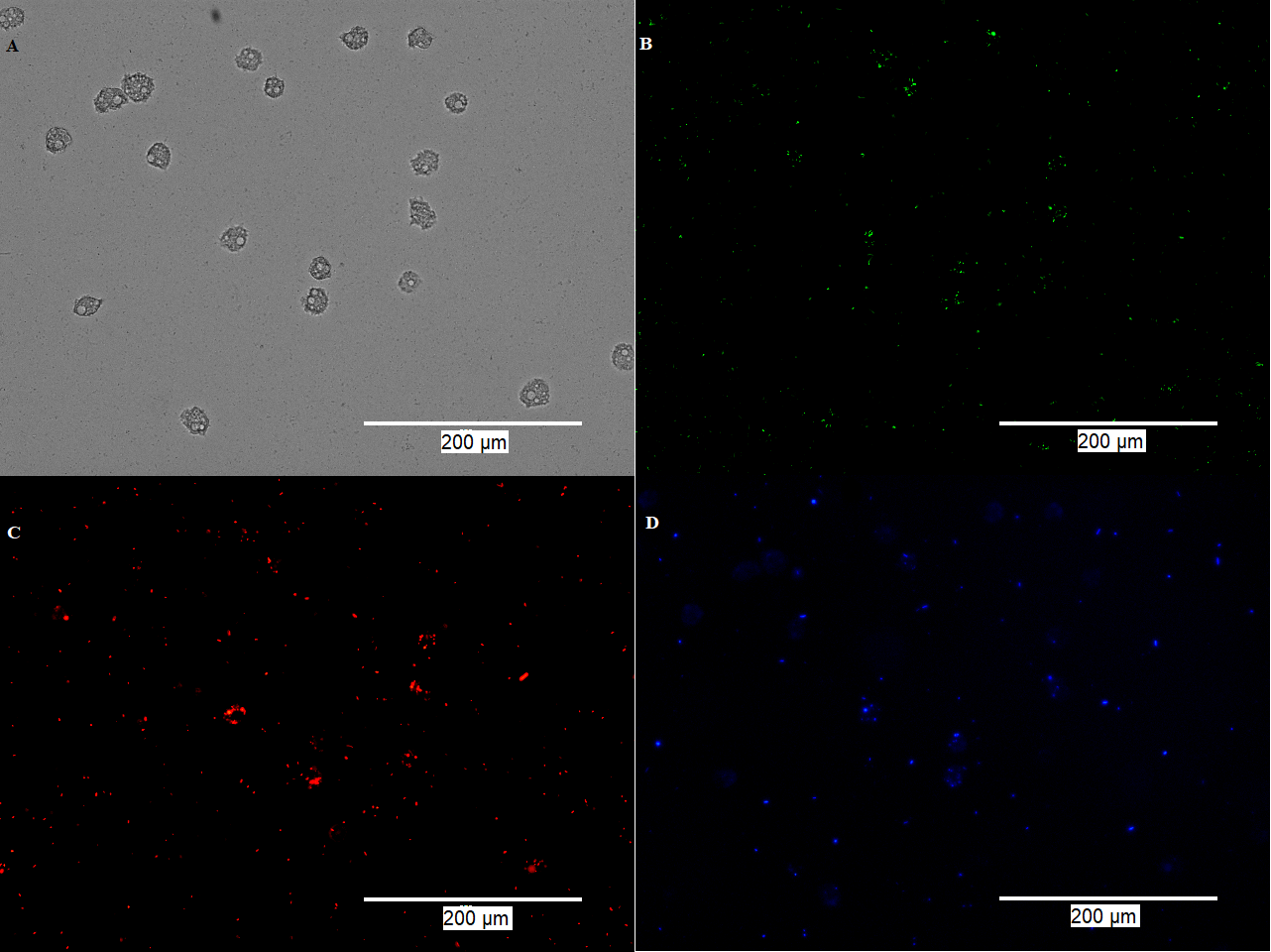


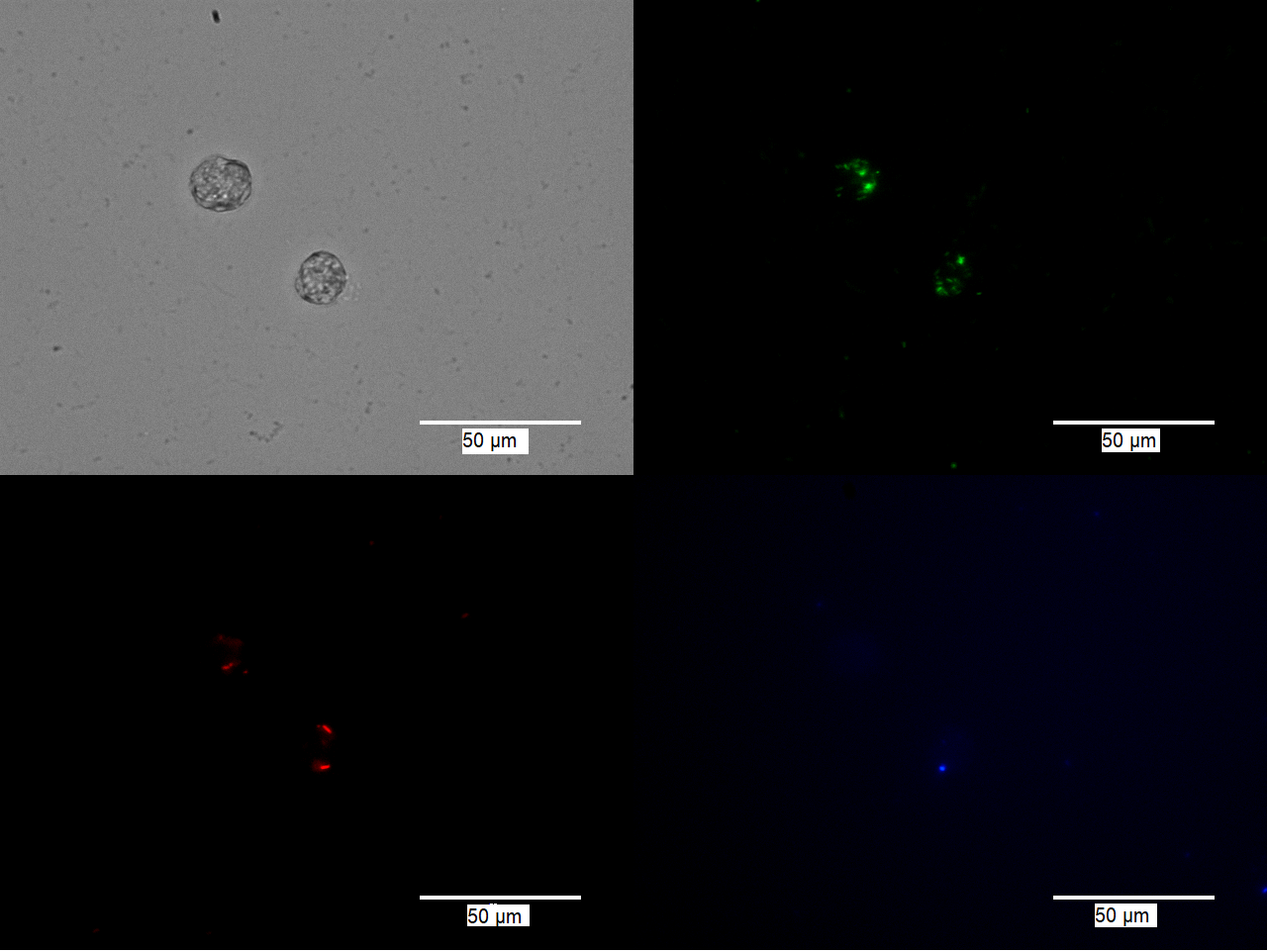


Figure S8. Preferential feeding on bacteria by *A. polyphaga* at RT at 2 h (Top) and 24 h (Bottom) of co-culture. The four images represent the same field of view under different fluorescent light channels to observe different bacteria. A. Mono-color transmission light channel for *A. polyphaga*, B. Green fluorescent *L. pneumophila* C. Texas-Red *E. coli* MG1655 D. DAPI channel for *E. coli* TOP10. The composite image (bottom) shows that *A. polyphaga* trophozoites phagocytose all the bacteria with equal preference for *L. pneumophila* and *E. coli*.


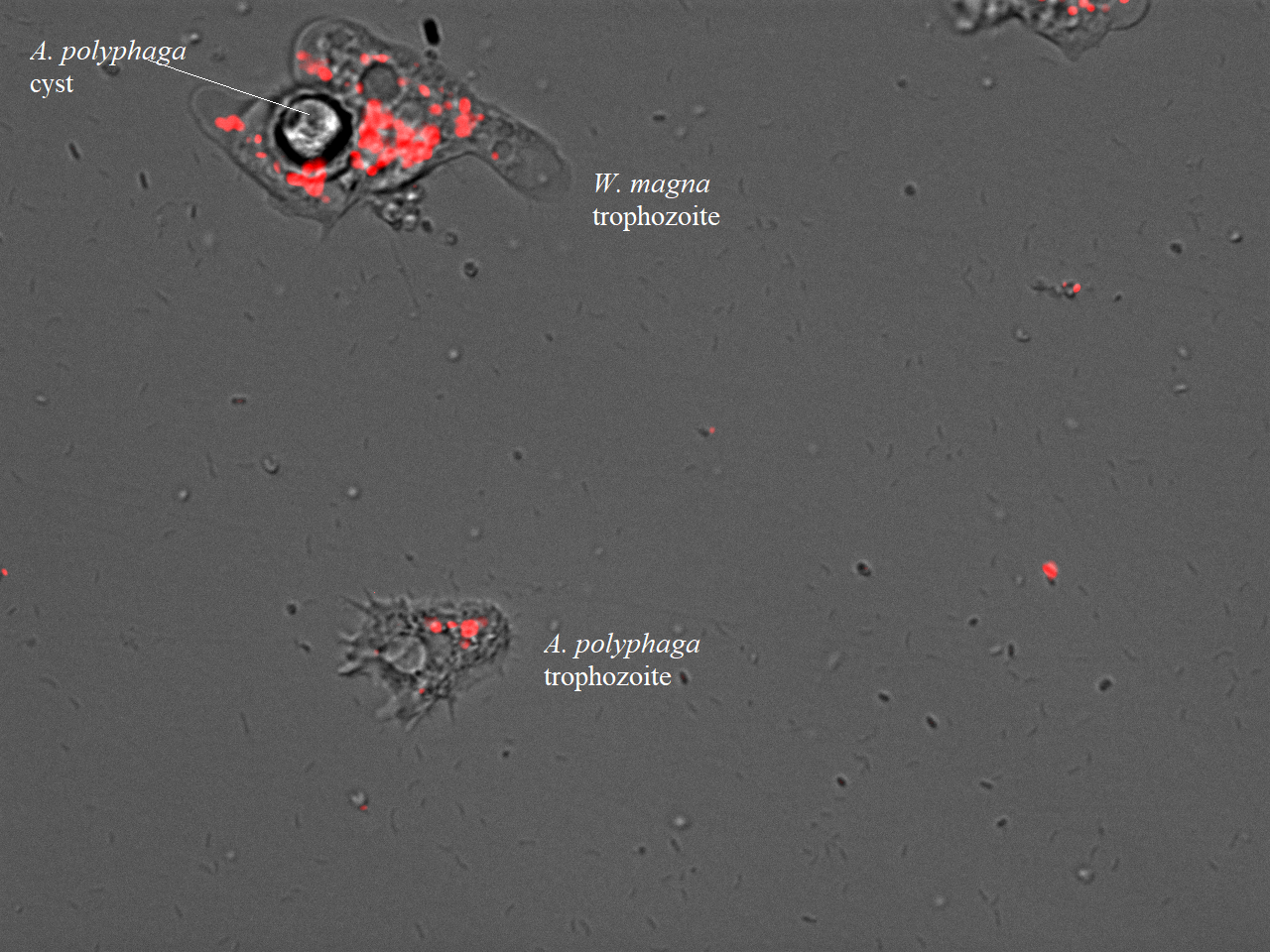


Video S9. Intracellular locations of *E. coli* MG1655 (red) in amoeba trophozoites (*W. magna* and *A. polyphaga*) and cyst (*A. polyphaga*). *W. magna* trophozoites was moving closely around the *A. polyphaga* trophozoites and cyst in water.


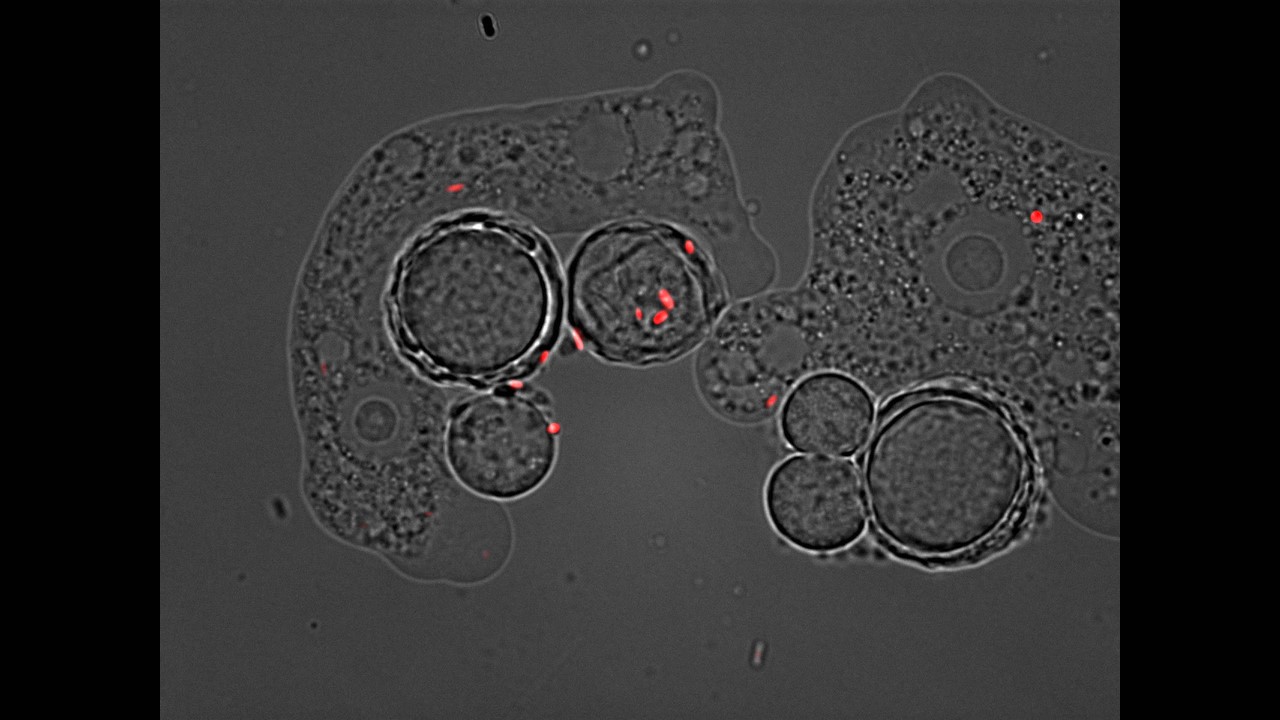


Video S10. Intracellular locations of *E. coli* MG1655 (red) in the amoeba cysts (*A. polyphaga* cysts were double layered and bigger, *V. vermiformis* cysts were smaller with smooth layer) at 96 h of co-culture. In *W. magna* trophozoites still some *E. coli* MG1655 cells left inside one of the food vacuoles.


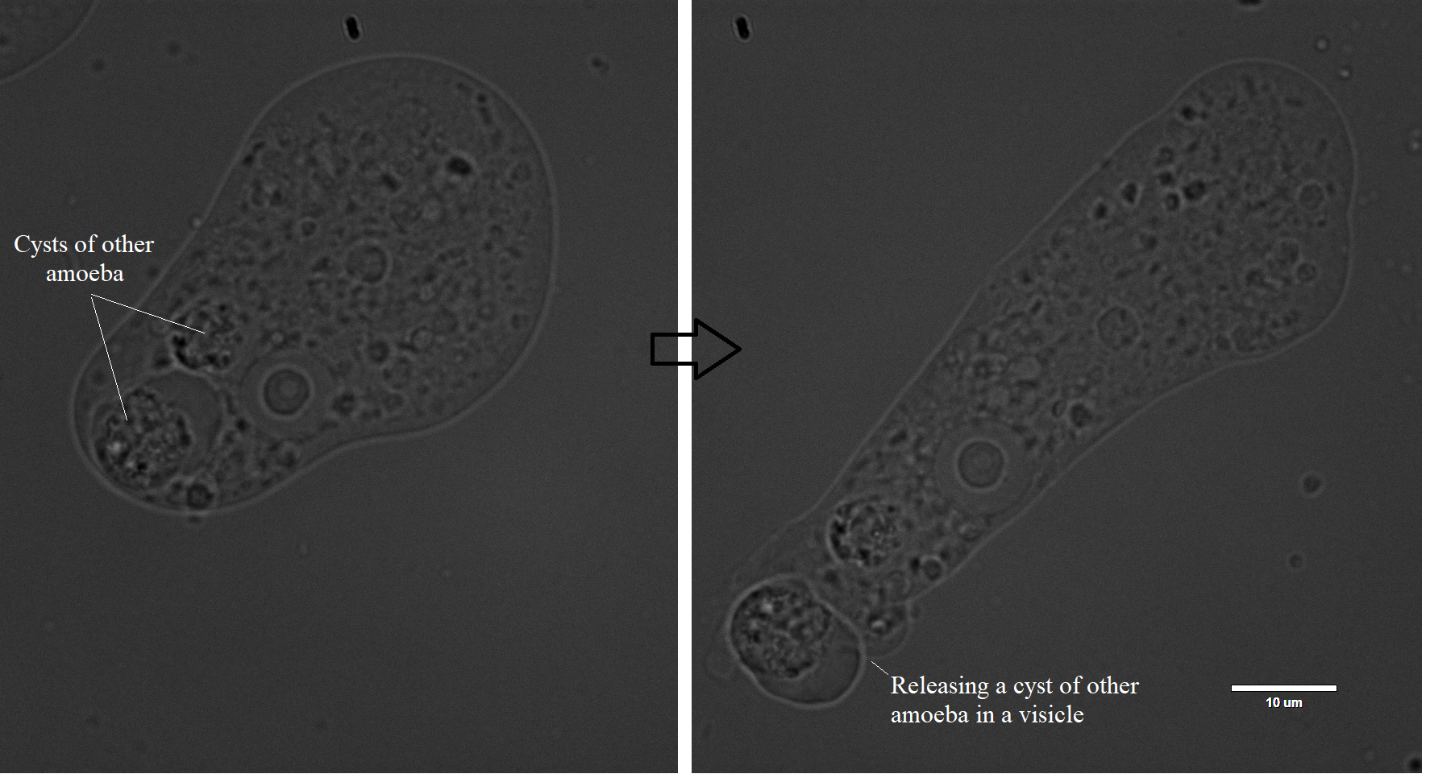


Figure S11. *W. magna* with cysts of other amoeba (*A. polyphaga* and *V. vermiformis* were present in the same culture) in a co-culture with *E. coli* (MG1655) in water (Left). Release of a cyst (possibly in a vesicle) of another amoeba from *W. magna* trophozoites (right).
